# Uncovering the Molecular Response of Oregano (*Origanum vulgare* L.) to ^12^C^6+^ Heavy-Ion Irradiation Through Transcriptomic and Metabolomic Analyses

**DOI:** 10.3390/cimb48010007

**Published:** 2025-12-21

**Authors:** Zhengwei Tan, Lei Li, Yan Liang, Chunming Li, Xiaoyu Su, Dandan Lu, Yao Sun, Lina Wang, Mengfan Su, Yiwen Cao, Huizhen Liang

**Affiliations:** 1 Institute of Chinese Herbal Medicines, Henan Academy of Agricultural Science, Zhengzhou 450002, China; zhwtan@126.com (Z.T.); 15136189572@163.com (L.L.); liangyanemail2024@126.com (Y.L.); lchm1212@163.com (C.L.); suxiaoyu_2014@163.com (X.S.); ludandan0710@163.com (D.L.); ruth_3912834@163.com (Y.S.); hnndlina@163.com (L.W.); sufancy1669@163.com (M.S.); yiwencao96@163.com (Y.C.); 2 Provincial Key Laboratory of Conservation and Utilization of Traditional Chinese Medicine Resources, Institute of Chinese Herbal Medicines, Henan Academy of Agricultural Sciences, Zhengzhou 450002, China

**Keywords:** *Origanum vulgare* L., plant mutagenesis breeding, secondary metabolites, gene expression profiling, terpenoid biosynthesis

## Abstract

*Origanum vulgare* L., a medicinal herb rich in bioactive phenols and terpenes, is recognized for its anti-inflammatory and antimicrobial properties. Heavy-ion beam mutagenesis, a sophisticated breeding technique, can induce significant variations in plants, thereby affecting their secondary metabolite production. This study utilized metabolomic and transcriptomic approaches to investigate the effects of ^12^C^6+^ heavy-ion irradiation on oregano. Our results indicated substantial changes in mutant lines, including marked alterations in plant height, leaf morphology, and biomass accumulation. Metabolomic analysis indicated that the differentially accumulated volatile compounds were primarily terpenoids. Furthermore, transcriptomic analysis indicated a predominant enrichment of differentially expressed genes in terpenoid biosynthesis. Integrated analyses identified key transcriptional changes in genes encoding terpenoid backbone enzymes, such as *GPPS*, *GGPPS*, *DXS*, and *HMGR*, and pinpointed candidate genes, including *TPS3*, *TPS6A*, *TPS6C*, *CYP71D178*, *CYP71D181*, and *CYP71D10B*, whose expression patterns were closely associated with the differential accumulation of carvacrol and thymol. This comprehensive study elucidates the molecular mechanisms underlying metabolic reprogramming induced by heavy-ion irradiation in oregano and offers valuable genetic resources for future metabolic engineering and precision breeding initiatives aimed at enhancing the production of valuable bioactive compounds.

## 1. Introduction

*Origanum vulgare* L., commonly known as oregano, is a perennial herb of the lamiaceae family with a long history of culinary and medicinal use across diverse cultures [1,2]. Modern pharmacological studies have substantiated its traditional applications, revealing that oregano extracts possess significant antioxidant, antibacterial, anti-inflammatory, antitumor, and insecticidal properties [3,4,5]. These broad-spectrum bioactivities are primarily attributed to its rich profile of secondary metabolites, particularly terpenoids, which are the most prominent bioactive constituents [6]. Numerous terpenoids exhibit substantial pharmacological potential, including anticancer, antimicrobial, anti-inflammatory, and antioxidant effects, as well as protective properties against neural damage [7,8]. The clinical relevance of terpenoids is exemplified by drugs such as artemisinin (a sesquiterpene) for malaria and paclitaxel (a diterpene) for cancers [9,10]. Within oregano, the predominant active terpenoids are monoterpenes and sesquiterpenes. In oregano, the principal active terpenoids are monoterpenes and sesquiterpenes. The phenolic monoterpenes thymol and carvacrol have been identified as the key bioactive and commercially valuable signature compounds, conferring the distinctive pungent aroma and forming the basis of the plant’s potent antimicrobial activity [11,12]. Consequently, the quality and commercial value of oregano are directly determined by the content and ratio of thymol and carvacrol. However, the molecular mechanisms governing their biosynthesis in oregano remain largely elusive. Therefore, elucidating the regulatory pathways of thymol and carvacrol synthesis is imperative to meet the growing demand for high-quality oregano.

In plants, the biosynthesis of terpenoids primarily relies on two key pathways: the mevalonate (MVA) pathway in the cytoplasm and the methylerythritol phosphate (MEP) pathway in plastids [13]. For monoterpenes, the precursors isopentenyl pyrophosphate (IPP) and its isomer dimethylallyl pyrophosphate (DMAPP) are primarily supplied by the MEP pathway. These two five-carbon units are condensed by geranyl pyrophosphate synthase (GPPS) to form geranyl pyrophosphate (GPP), the universal carbon skeleton for monoterpenes. GPP is subsequently cyclized by specific terpene synthases (TPSs) to generate diverse monoterpene skeletons [14]. In Lamiaceae plants, thymol and carvacrol are biosynthesized from γ-terpinene through sequential oxidation by cytochrome P450 monooxygenases (P450s) of the CYP71D subfamily and dehydrogenation by a short-chain dehydrogenase/reductase (SDR), culminating in aromatic phenols via keto-enol tautomerism [15]. The entire biosynthetic pathway is subject to multi-layered regulation. Beyond the expression of structural genes (e.g., *GPPS*, *TPS*, and *CYP71D*), the pathway is finely tuned by various transcription factors (TFs). In other plant species, transcription factor (TF) families, including bZIP, TCP, MYB, and bHLH, have been shown to regulate terpenoid biosynthesis [16,17,18,19]. Given that alterations in the expression or activity of any these regulatory or structural genes can critically influence metabolic flux and end product accumulation, a comprehensive understanding of this regulatory network is essential.

Genetic diversity provides the essential foundation for variety improvement and germplasm innovation, directly influencing a population’s adaptive and evolutionary capacity [20,21]. Among methods to generate such diversity, heavy-ion beam irradiation is a promising physical mutagenesis technique. It boasts unique advantages such as low physiological damage, a high mutation rate, a broad mutation spectrum, and rapid stabilization of mutant traits [22]. At the molecular level, this mutagenesis primarily generates large-fragment deletions, insertions, and chromosomal structural variations [23]. These mutations are particularly impactful, as they often cause complete loss of gene function or critical amino acid changes, effectively disrupting rate-limiting steps and reprogramming the regulatory networks of secondary metabolic pathways to induce significant phenotypic alterations [24,25]. The application of heavy-ion beam irradiation has been demonstrated to be an effective method of inducing functional mutations while concurrently maintaining high survival rates. This combination renders it a potent instrument for germplasm innovation and gene discovery in the domain of plant breeding, including that of medicinal species [26,27,28]. However, the application of this potent technique to oregano breeding has not been documented.

In this study, headspace solid-phase microextraction (HS-SPME) coupled with gas chromatography–tandem mass spectrometry (GC-MS) and transcriptome sequencing (RNA-Seq) were employed to analyze DAVs and DEGs dynamic changes to ^12^C^6+^ heavy-ion beam irradiation for *Origanum vulgare* L. and to elucidate the molecular mechanisms underlying the biosynthesis of terpenoids, including thymol and carvacrol. These findings provide new insights into the molecular basis of heavy-ion mutagenesis and offer practical genetic resources for the targeted improvement of oregano.

## 2. Materials and Methods

### 2.1. Plant Materials

Seeds of oregano (*Origanum vulgare* L.) were chosen as the experimental material. A total of 1500 seeds were utilized for a dose-finding study. These seeds were exposed to a gradient of ^12^C^6+^ heavy-ion irradiation doses(0, 30, 60, 90,120 and 140 Gray (Gy)), with the non-irradiated seeds (0 Gy) serving as the control group. The germination rate, seedling emergence rate, and seedling survival rate were calculated for each dose. The semi-lethal radiation dose (LD_50_) was determined from this data (60 Gy) and was selected as the optimal dose for large-scale mutagenesis. Subsequently, a separate batch of 500 seeds was irradiated in a single session at this predetermined dose to establish the mutant population. 500 mutagenized seeds were cultivated as T0 plants in xian County, where plants were self-pollinated to advance the generations. This process of self-pollination continued to yield homozygous mutants. Subsequently, twenty seeds from each T2 line were selected and planted at the Henan Academy of Agricultural Sciences experimental station in Xinxiang City for cultivation. From this T2 population, homozygous lines exhibiting typical mutant traits were identified, screened, and selected for use in subsequent experiments.

### 2.2. Assessment of Morphological Traits

The growth status of oregano plants from the three groups was documented in the field through photographic records. Morphological evaluations were performed during the growth to seedling stage. Plant height, measured from apex to base, was determined using a standard ruler. The dimensions of the largest leaf, including maximum length and width, were assessed through image analysis utilizing ImageJ software (version: 1.54g). Additionally, the average fresh weights of the leaves and stems were recorded using an analytical balance.

### 2.3. Measurement of Soil and Plant Analyzer Development (SPAD)

After reaching the seedling stage, the chlorophyll content of the leaves in the three plant groups was measured using a handheld portable chlorophyll meter (Spad-502 plus, Konica Minolta, Inc., Osaka, Japan). Two measurements were obtained from each of the two largest pairs of opposite leaves per plant. Each sample was evaluated with a minimum of three biological replicates.

### 2.4. GC-MS/MS Analysis of Volatile Compounds in Oregano Leaves

Extract analysis and metabolic identification were conducted by MetWare (Wuhan, China) according to standard procedures. The total ion currents (TICs) of QC specimens are presented in Figure S2. The variable importance of projection (VIP) score of the application (O) partial least squares model was applied to fit the differentiated metabolites in comparison groups. Metabolites with FC ≥ 1 and VIP ≥ 1 are considered to be differentially accumulated metabolites. Metabolite data analysis was conducted using the MetaboAnalyst (version 6.0). Furthermore, the PCA method was used to calculate the variability in various groups, and functional annotation of DAVs was conducted via the KEGG pathway.

### 2.5. Transcriptome Sequencing and Analysis

Total RNA was extracted from 0.1 g of oregano leaves using a Plant Total RNA Extraction Kit (Omega Bio-Tek, Norcross, GA, USA). For each group, sequencing libraries were constructed from three independent biological replicates. Subsequently, a total of 4 μg of RNA per sample was used for library preparation, and the cDNA libraries were sequenced on the Illumina NovaSeq X Plus platform (version 1.3.1, San Diego, CA, USA). Raw reads were processed with fastp (v0.20.0) to obtain clean reads by removing adapter sequences, reads with >10% unknown bases (N), poly-A reads, and low-quality reads (where over 50% of the bases had a Phred score ≤ 20). De novo transcriptome assembly was performed using Trinity (v2.8.5) with default parameters. The assembly quality was assessed using the N50 statistic and BUSCO. The assembled unigenes were functionally annotated by aligning them against the NR (https://ftp.ncbi.nih.gov/blast/db, accessed on 12 December 2025), Swiss-Prot (https://www.expasy.org/resources/uniprotkb-swiss-prot, accessed on 12 December 2025), KEGG (https://www.genome.jp/kegg), and COG/KOG (https://www.ncbi.nlm.nih.gov/research/COG, accessed on 12 December 2025) databases using BLASTx (version 2.17.0+) with an E-value cutoff of 1 × 10^−5^. Protein domains were annotated using Pfam_Scan (version 1.6) against the Pfam database. For plant samples, transcription factors were identified using hmmscan against PlantTFDB (version 5.0) and BLASTp (version 2.17.0+) against PRGdb (version 4.0), respectively.

Sample relationships were assessed by Principal Component Analysis (PCA) and Pearson correlation heatmaps based on the gene expression matrix. We standardized the raw transcriptomic expression matrix (counts) using DESeq2 (v1.26.0) and perform differential expression analysis. Genes with a false discovery rate (FDR) < 0.05 and an absolute log_2_FC (fold change) > 1 were considered significantly DEGs. Use RSEM (v1.3.0) to convert raw count data to FPKM values.

Gene Ontology (GO) and Kyoto Encyclopedia of Genes and Genomes (KEGG) pathway enrichment analyses of the differentially expressed genes (DEGs) were performed using the clusterProfiler package (version 4.12). GO terms or KEGG pathways with a corrected *p*-value (Bonferroni for GO, FDR for KEGG) ≤ 0.05 were considered significantly enriched.

### 2.6. Real-Time Quantitative PCR (RT-qPCR) Analysis for Gene Expression

Total RNA was extracted from plant tissues utilizing the Plant RNA Kit (Omega Bio-Tek, USA). The cDNA template was synthesized with HiScript III All-in-one RT SuperMix Perfect for qPCR (+gDNA wiper) (Vazyme, Nanjing, China). The expression levels of target genes were assessed using a StepOnePlus^TM^ Detection System (Applied Biosystems, Foster City, CA, USA). *EF-1α* served as a control for normalizing the expression data. Primers were designed using Primer Premier 5 and are detailed in Supplementary Table S8. Relative mRNA expression levels were calculated according to the 2^−ΔΔCt^ method [29].

### 2.7. Statistical Analysis

In this study, GraphPad Prism (v10.3.0) was used to analyze the significance of differences between the data and to plot the bar graphs. The means were compared by one-way ANOVA and Duncan’s multiple range test at the 5% significance level.

## 3. Results

### 3.1. Generation of Heavy-Ion Irradiated Mutants and Phenotypic Characterization of NZ1 and NZ2 in Origanum vulgare *L.*

The success of radiation mutagenesis breeding hinges on identifying the appropriate radiation dose, necessitating a thorough evaluation of both plant survival rates and the likelihood of obtaining desirable mutant traits. Currently, the LD50 (median lethal dose) serves as a reference standard, often regarded as the dose that yields optimal mutagenic effects [30]. To explore the effects of heavy-ion irradiation on oregano and to establish a theoretical foundation for heavy-ion beam irradiation mutagenesis breeding, dry oregano seeds were exposed to ^12^C^6+^ heavy ions at doses of 0, 30, 60, 90, 120, and 140 Gy. Measurements of germination rates, emergence rates, and survival rates were conducted to determine the optimal radiation dose for mutagenesis. The results are presented in Figure S1. Compared to the wild type (WT), the application of ^12^C^6+^ irradiation at doses from 0 to 90 Gy had a negligible effect on the germination rate of oregano. In contrast, doses of 120 and 140 Gy led to a significant decrease in this rate. Additionally, the seedling emergence rate showed a pronounced decline in response to increased irradiation doses. The emergence rate decreased by approximately 5% at 10 Gy compared to the control (CK), while doses of 30, 60, and 100 Gy resulted in a reduction of about 50% in comparison with CK. Treatment with 140 Gy led to an emergence rate of only 5%. The survival rate for CK stood at 82.60%, while rates for the 10, 30, 60, 120, and 140 Gy irradiation treatments were 70.91%, 64.82%, 38.65%, 13.14%, and 0%, respectively. Notably, all seedlings subjected to the 140 Gy treatment perished within the designated observation period. Based on the findings, it can be inferred that the LD50 is 60 Gy.

**Figure 1:**
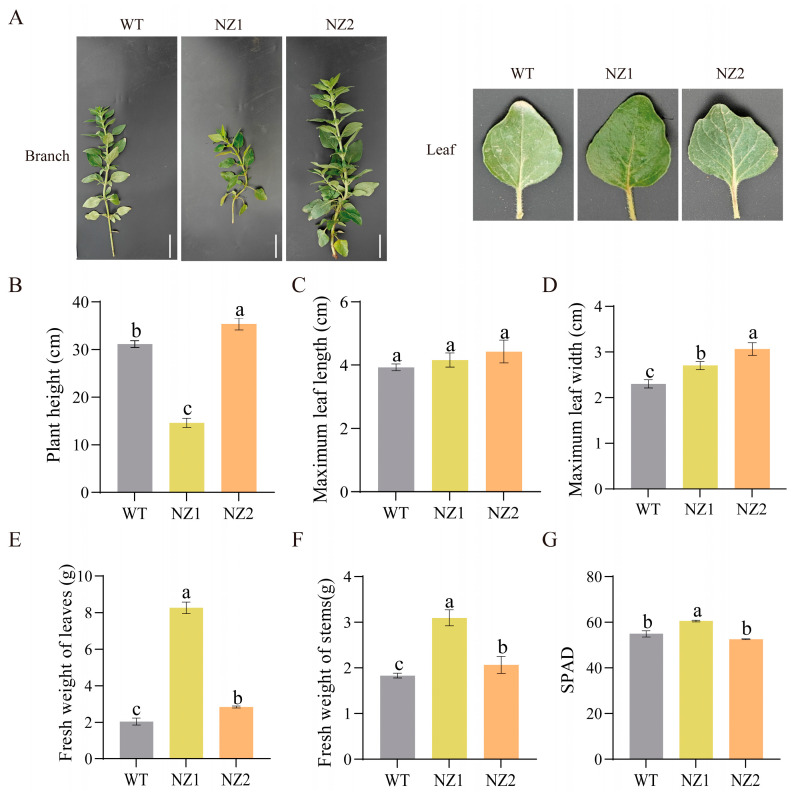
Effect of ^12^C^6+^ heavy iron irradiation on growth characteristics of oregano. (**A**) Phenotypes of branch and leaves in oregano mutants NZ1 (**middle**), NZ2 (**right**), and WT (**left**); (**B**–**G**) plant height, maximum leaf length, maximum leaf width, fresh weight of leaves, fresh weight of stems, and SPAD. Bar = 5 cm. Different letters indicate significant differences between groups at *p* < 0.05 according to Duncan’s multiple range test.

Seeds irradiated with a 60 Gy dose of ^12^C^6+^ for one hour were subsequently selected for screening. After three consecutive years of self-pollination and cultivation, several potential homozygous oregano mutant lines were obtained. The following parameters were measured for the mutants: plant height, maximum leaf length, maximum leaf width, leaf fresh weight, stem fresh weight, and SPAD. Mutants NZ1 and NZ2 were identified as exhibiting distinct mutant characteristics. The results are shown in Figure 1; compared to the mean plant height of the WT at 31.16 cm, the mean plant heights of NZ1 and NZ2 were significantly decreased by 16.57 cm and significantly increased by 4.17 cm, respectively. Furthermore, an increase in the average maximum leaf width of NZ1 and NZ2 was recorded, with measurements of 0.30 cm and 0.76 cm, respectively, compared to the WT at 2.30 cm. This study investigated the effects of heavy-ion irradiation on the fresh weights of the leaves and stems of NZ1 and NZ2, revealing that both samples exhibited similar responses to the treatment. Relative to the mean leaf fresh weight of the control group (2.03 g), NZ1 and NZ2 demonstrated increases of 6.23 g and 0.80 g, respectively. In terms of stem fresh weight, compared to the control group’s mean (1.83 g), NZ1 and NZ2 showed increases of 1.27 g and 0.23 g, respectively. The SPAD for NZ1 was significantly higher than those recorded for both WT and NZ2. The development and characterization of variants NZ1 and NZ2 provide a basis for future comprehensive research.

### 3.2. Metabolomics Profiling of Volatile Compounds in Heavy-Ion-Induced Oregano Mutants (NZ1, NZ2) and the Wild-Type

**Figure 2:**
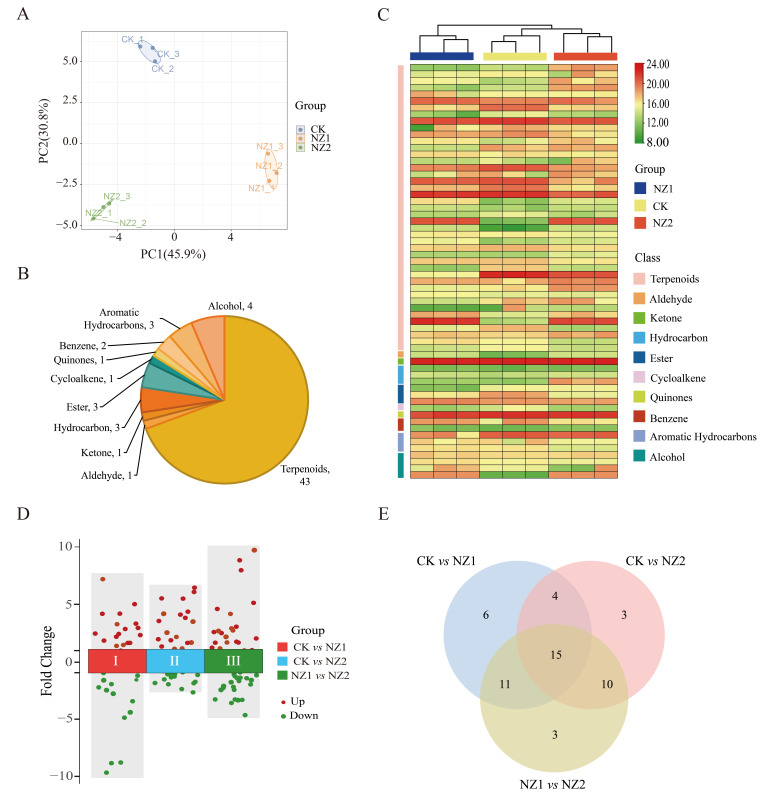
Metabolite analysis of volatile compounds in heavy-ion-induced oregano mutants (NZ1, NZ2) and the wild-type plant. (**A**) Principal component analysis (PCA) of all 9 samples. (**B**) Classification and proportion of VOCs detected in the oregano leaves. (**C**) Heatmap analysis of all VOCs in three plants. (**D**) Volcano plots of DAVs in various comparison groups. (**E**) Venn diagram of DAVs.

The economic value of oregano is primarily determined by its essential oil yield, which consists mainly of phenolic monoterpenes, including carvacrol and thymol, along with their precursors. To analyze the volatile organic compounds in the leaves of the homozygous lines NZ1 and NZ2, generated through heavy-ion mutagenesis, as well as their WT counterpart, headspace solid-phase microextraction coupled with gas chromatography-tandem mass spectrometry (HS-SPME-GC-MS/MS) was employed. Pearson correlation analysis indicated strong correlations among biological replicates within the same line, while revealing weak correlations between different lines) (Figure S3). Principal component analysis (PCA) of the detected volatiles (Figure 2A) showed that the first two principal components accounted for 45.9% and 30.8% of the variance, respectively. Notably, NZ1, NZ2, and WT were distinctly separated, and the three biological replicates of each sample were in close proximity, thereby confirming the reliability of the experimental results and highlighting the significant disparities among the samples.

A total of 62 volatile organic compounds were identified across the three materials and classified into 10 categories: terpenoids, aldehydes, ketones, hydrocarbons, esters, cycloalkenes, quinones, benzenes, aromatic hydrocarbons, and alcohols (Table S1). The analysis indicated that terpenoids were the most abundant volatile organic compounds, totaling 43 spcies. These were followed by alcohol (4), hydrocarbons (3), esters (3), aromatic hydrocarbons (3), benzenes (2), aldehydes (1), ketone (1), cycloalkene (1), and quinones (1) (Figure 2B). Additionally, a heatmap illustrating the relative content of volatile metabolites (Figure 2C) revealed that the three materials clustered into three distinct branches. The biological replicate samples of each variety were grouped together, highlighting clear differences among the materials, which aligns with the PCA results.

A comparison among three materials was conducted to further investigate the impact of heavy-ion irradiation on volatile organic compounds in oregano leaves. In the comparisons between WT vs. NZ1, WT vs. NZ2, and NZ1 vs. NZ2, 36, 32, and 39 differentially accumulated volatiles (DAVs) were, respectively, identified (Figure 2D). Across the three comparative analyses, 20 DAVs were up-regulated and 16 were down-regulated in WT vs. NZ1, 12 DAVs were up-regulated and 20 were down-regulated in WT vs. NZ2, and 17 DAVs were up-regulated and 22 were down-regulated in NZ1 vs. NZ2 (Figure 2E). Notably, 15 metabolites were identified as common DAVs in all three comparison groups. Analysis using the KEGG pathway revealed enrichment of these identified DAVs in biosynthetic pathways, particularly those associated with terpenoid synthesis. In conclusion, the results indicate significant variations in metabolites among the three materials, highlighting the substantial impact of heavy-ion irradiation on the volatile metabolic profile of oregano, particularly affecting terpenoid compounds.

### 3.3. Transcriptome Analysis Reveals Dynamic Changes in Gene Expression in Heavy-Ion Induced Oregano Mutants (NZ1, NZ2) and the Wild-Type (WT)

**Figure 3:**
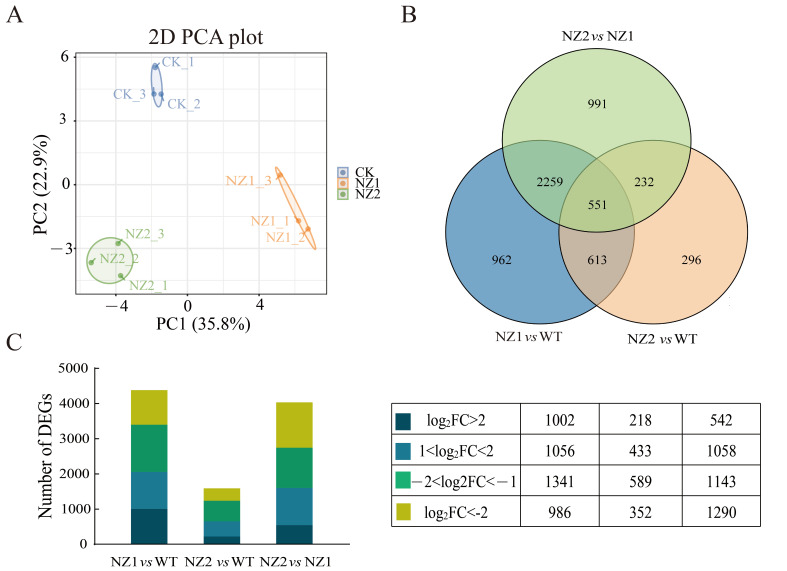
Characterization of DEGs in oregano NZ1, NZ2 and WT. (**A**) PCA of 9 samples. (**B**) Venn diagram of DEGs. (**C**) Statistics of DEGs in NZ1, NZ2, and WT.

To further elucidate the effects of heavy-ion irradiation on gene expression in oregano leaves, a de novo transcriptome analysis was performed using RNA sequencing technology on leaves from NZ1, NZ2, and WT. The construction of nine libraries yielded 53.45 GB of clean data (Table S2), with average Q20, Q30, and GC content values of 98.12%, 94.88%, and 49.14%, respectively. Principal component analysis (PCA) of the nine oregano samples (Figure 3A) demonstrated that the first two principal components (PC1 and PC2) accounted for 35.8% and 22.9% of the variance, respectively. The samples clustered into three distinct groups, indicating high reproducibility within groups and significant differences between them. This validates their suitability for subsequent analysis. The clean data from the nine RNA-seq samples were assembled using Trinity software (version 2.15.2), resulting in the identification of 41,492 genes. These genes were annotated against the Nr, SwissProt, KEGG, and COG/KOG databases, yielding 26,183, 19,175, 25,551, and 15,225 annotated genes, respectively. Subsequently, three comparison groups (NZ1 vs. WT, NZ2 vs. WT, and NZ2 vs. NZ1) were employed to identify differentially expressed genes (DEGs) induced by heavy-ion irradiation. The comparisons revealed 4385 DEGs (2058 up-regulated and 2327 down-regulated), 1592 DEGs (651 up-regulated and 941 down-regulated), and 4033 DEGs (1600 up-regulated and 2433 down-regulated), as shown in Figure 3B,C (Tables S3–S5). Notably, out of the total 6204 DEGs identified, 551 were common across all three comparison groups.

### 3.4. GO Annotation and KEGG Enrichment Analysis of DEGs

To elucidate the impact of heavy-ion irradiation, particularly the deposition of ^12^C^6+^ ions, on gene expression in oregano, we conducted Gene Ontology (GO) annotation analysis on the DEGs identified from the relevant pathways. The outcomes are presented in Figure S4. In the comparison between NZ1 and WT, the DEGs were predominantly associated with molecular function, biological process, and cellular component categories. Notably, under the Molecular Function category, the terms Binding (1815 genes, representing 41.39% of total DEGs) and Catalytic Activity (1501 genes, 34.23%) stood out. In the Cellular Component category, Cellular Anatomical Entity (1286 genes, 29.33%) exhibited significance. Regarding Biological Process, Cellular Process (1772 genes, 40.41%) and Metabolic Process (1598 genes, 36.44%) encompassed a substantial portion of the annotated genes. In the comparison between NZ2 and WT, DEGs were annotated according to Molecular Function and Biological Process. Notably, the terms Binding (629 genes, 39.51%) and Catalytic Activity (524 genes, 32.91%) were predominant in Molecular Function, while Cellular Process (565 genes, 35.49%) and Metabolic Process (510 genes, 32.04%) were highly represented in Biological Process. In the NZ2 versus NZ1 comparison, DEGs were annotated across Molecular Function, Biological Process, and Cellular Component. The most frequently annotated terms included Binding (1644 genes, 40.76%) and Catalytic Activity (1355 genes, 33.60%) in Molecular Function; Cellular Process (1589 genes, 39.40%) and Metabolic Process (1451 genes, 36.00%) in Biological Process; and Cellular Anatomical Entity (1142 genes, 28.32%) in Cellular Component.

**Figure 4:**
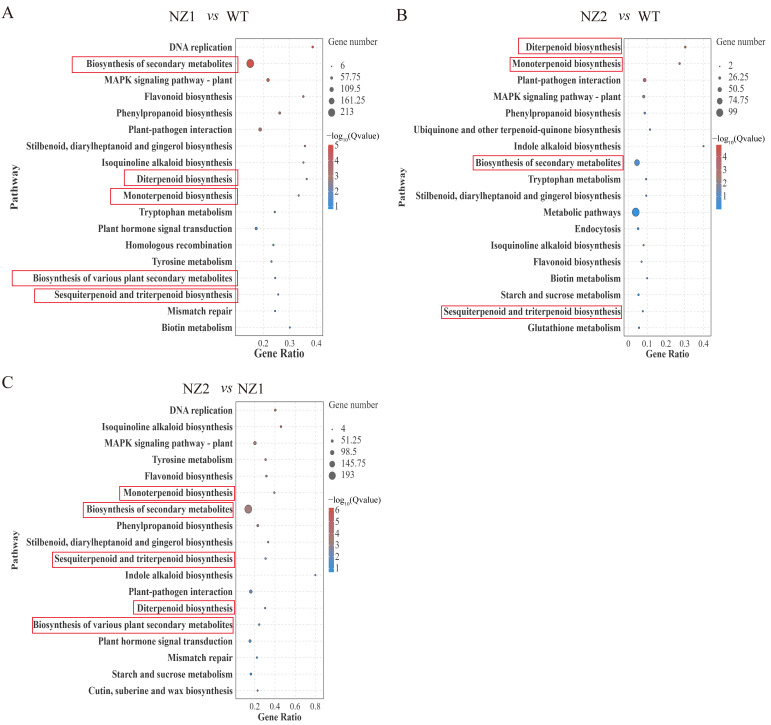
KEGG enrichment of DEGs in NZ1 vs. WT (**A**), NZ2 vs. WT (**B**), and NZ2 vs. NZ1 (**C**), respectively.

KEGG pathway analysis was performed on the DEGs identified from all three comparison groups, revealing their association with various metabolic pathways. Screening the top 18 pathways based on FDR value showed that 10 metabolic pathways were common across the three comparison groups (Figure 4). Processes impacted by heavy-ion irradiation included biosynthesis of secondary metabolites, MAPK signaling pathway, flavonoid biosynthesis, phenylpropanoid biosynthesis, plant–pathogen interaction, stilbenoid, diarylheptanoid, and gingerol biosynthesis, isoquinoline alkaloid biosynthesis, diterpenoid biosynthesis, monoterpenoid biosynthesis, tryptophan metabolism, plant secondary metabolism, and sesquiterpenoid and triterpenoid biosynthesis. These results highlight the significant influence of heavy-ion irradiation on various physiological processes in the two selected typical mutants, particularly those related to terpenoids’ synthesis and metabolism among secondary metabolites.

### 3.5. Analysis of Transcription Factors Among DEGs

**Figure 5:**
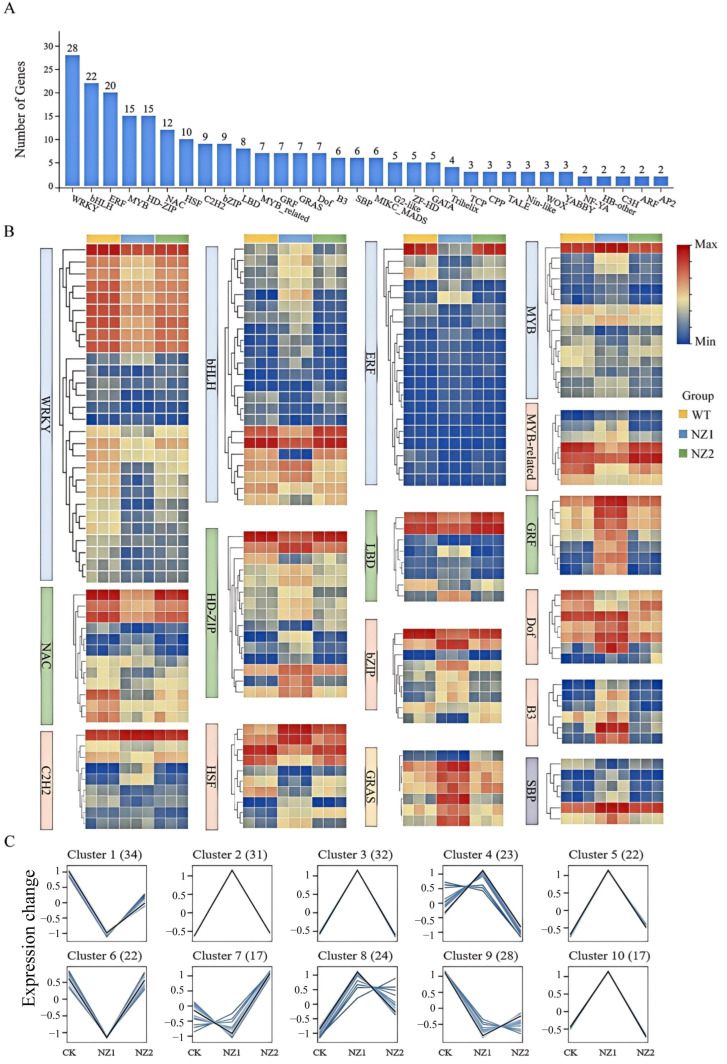
Differential expression analysis of transcription factors in NZ1, NZ2, and WT. (**A**) Statistical analysis of transcription factors in DEGs. (**B**) Heat map of expression levels of 16 transcription factor families. (**C**) K-means clustering analysis of 250 differential expression of transcription factors.

Plant transcription factors (TFs) are essential regulatory proteins that precisely control the transcriptional expression levels of target genes by binding specifically to their promoter regions. These proteins are crucial in various biological processes, including plant growth and development, stress responses, and the synthesis of secondary metabolites. To identify TFs potentially involved in heavy-ion-induced metabolic reprogramming, we first screened all differentially expressed genes (DEGs) from the comparisons of NZ1 vs. WT, NZ2 vs. WT, and NZ2 vs. NZ1 for TF annotation. This identified a total of 250 TFs derived from the combined DEG sets, belonging to 32 distinct families (Figure 5A). Comparing the two mutant lines separately to the wild-type, we observed distinct TF family profiles: NZ1 showed prominent changes in WRKY (28), bHLH (22), and ERF (20) families, while NZ2 was marked by alterations in MYB (15), HD-ZIP (15), and NAC (12) families, among others (Figure 5B).

To further uncover expression patterns associated with terpenoid accumulation, K-means clustering was performed on the 250 TFs across the three genotypes (WT, NZ1, NZ2). The TFs were grouped into 10 clusters (Figure 5C), comprising 34, 31, 32, 23, 22, 22, 17, 24, 28, and 17 TFs, respectively (Table S6). Notably, correlating with the content of carvacrol and thymol in these materials, it is suggested that TFs in Cluster 1 and Cluster 6 may play a role in the synthesis of carvacrol, while those in Cluster 9 may be linked to the synthesis of thymol.

### 3.6. Analysis of DEGs Involved in the Terpenoids Biosynthesis Pathways in Origanum vulgare *L.*

**Figure 6:**
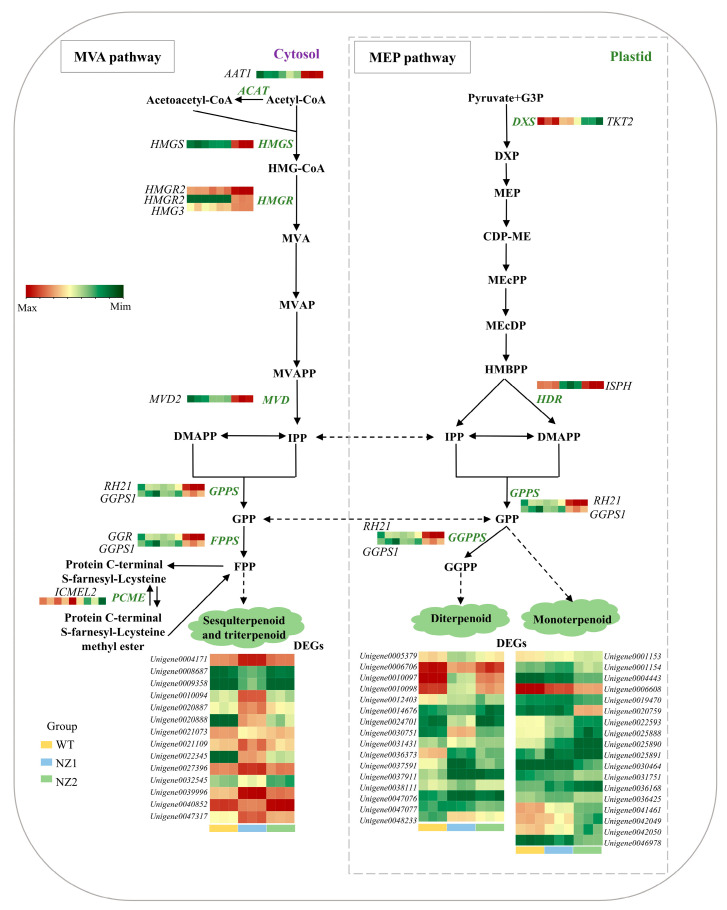
Profiles of genes encoding enzymes involved in terpenoids biosynthesis. The left and right showed the MVA and MEP pathways in oregano, respectively. Key enzymes in DEGs include acetyl-CoA C-acetyltransferase (*AACT*), hydroxymethylglutaryl-CoA synthase (*HMGS*), hydroxymethylglutaryl-CoA reductase (*HMGR*), mevalonate-5-diphosphate decarboxylase (*MVD*), geranyl pyrophosphate synthase (*GPPS*), farnesyl pyrophosphate synthase (*FPPS*), 1-deoxy-D-xylulose-5-phosphate synthase (*DXS*), and geranylgeranyl pyrophosphate synthase (*GGPPS*). The color scale from green to red represents the abundance of DEGs ranging from low to high levels in WT, NZ1, and NZ2.

In plants, terpenoid compounds are synthesized via two distinct metabolic pathways: the mevalonate (MVA) pathway and the methylerythritol phosphate (MEP) pathway. To further investigate the molecular mechanisms underlying terpenoid biosynthesis in oregano leaves, we analyzed DEGs from the transcriptome. This analysis identified a total of 59 genes encoding enzymes associated with terpenoid biosynthesis that exhibited differential expression (Figure 6). Within the terpene backbone synthesis pathway, several genes in the MEP pathway exhibited notable sensitivity to heavy-ion irradiation, including *DXS* (*Unigene0010384*), *HDR* (*Unigene0035540*), *GPPS* (*Unigene0004264*, *Unigene0044469*), and *GGPPS* (*Unigene0004264*, *Unigene0044469*). Additionally, in the MVA pathway, genes such as *ACAT* (*Unigene0039294*), *HMGS* (*Unigene0042224*), *HMGR* (*Unigene0031242*, *Unigene0047434*, *Unigene0016941*), *MVD* (*Unigene0012560*), *GPPS* (*Unigene0004264*, *Unigene0044469*), *GGPPS* (*Unigene0004264*, *Unigene0044469*), and *PCME* (*Unigene0034971*) also exhibited significant changes in expression in response to the irradiation treatment (Table S7).

### 3.7. Identification of OvTPSs and OvCYP71Ds in the Thymol and Carvacrol Biosynthesis Pathway in Origanum vulgare *L.*

**Figure 7:**
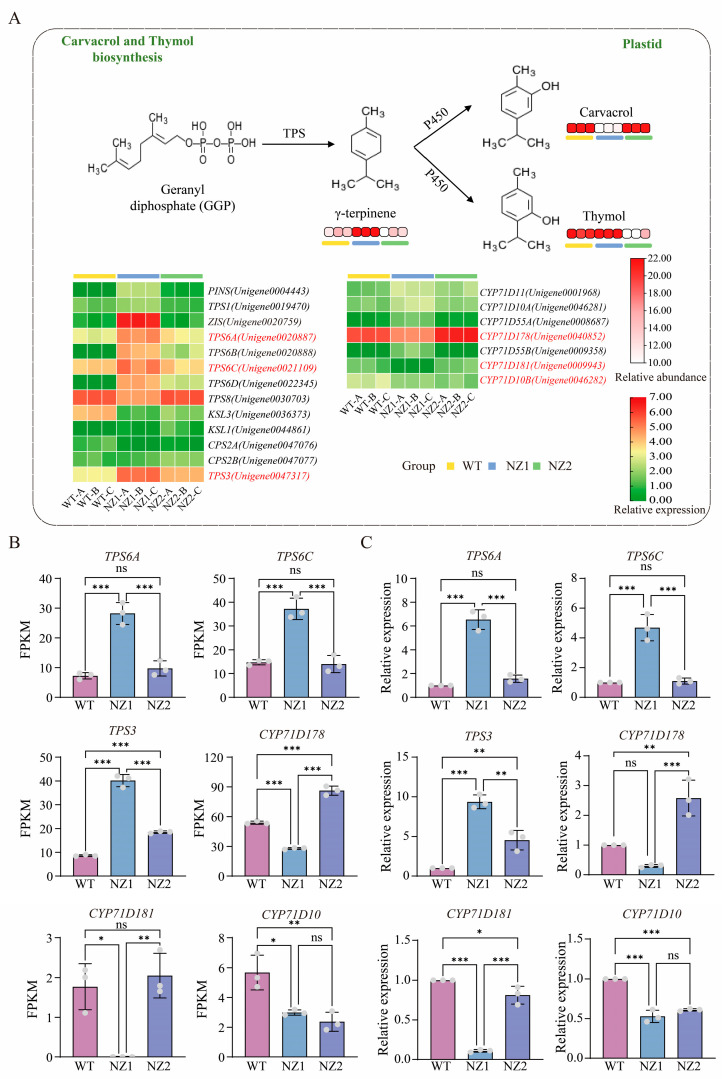
DEGs and DAVs involved in carvacrol and thymol biosynthesis. (**A**) Scheme of carvacrol and thymol biosynthesis in oregano. (**B**,**C**) Validation of *OvTPSs* and *OvCYP71Ds* using RT-qPCR. The data are presented as mean values ± SD, and statistical significance was analyzed using a one-way ANOVA. (* *p* < 0.05, ** *p* < 0.01, *** *p* < 0.001, ns: not significant).

In oregano, the compounds thymol and carvacrol are synthesized via the monoterpenoid biosynthesis pathway. Terpene synthases (TPSs) serve as crucial enzymes in terpenoid biosynthesis, facilitating the final step in the formation of the terpenoid backbone. During monoterpene synthesis, γ-terpinene, produced through TPS catalysis, undergoes a hydroxylation reaction regulated by regioselective cytochrome P450 hydroxylases (*CYP450s*). This reaction introduces a hydroxyl group at distinct positions (C3 or C2) on the aromatic ring, resulting in the specific formation of carvacrol or thymol, respectively. In this study, the annotation and analysis of transcriptome results resulted in the identification of 13 *TPSs* and 7 *CYP71Ds *(Figure 7A). Notably, among the identified *TPSs* and *CYP71Ds*, the expression patterns of *TPS6A* (*Unigene0020887*), *TPS6C* (*Unigene0021109*), and *TPS3* (*Unigene0047317*) at the transcriptional level aligned with the trend in γ-terpinene content observed in the oregano samples. Additionally, *CYP71D178* (*Unigene0040852*) and *CYP71D181* (*Unigene0009943*) displayed expression patterns that matched the accumulation trend of carvacrol, while *CYP71D10B* (*Unigene0046282*) corresponded to the trend in thymol content (Figure 7B,C). These findings indicate that these genes may facilitate the biosynthesis and accumulation of terpenoid compounds—particularly carvacrol and thymol—in oregano through positive regulation, thereby enhancing the levels of bioactive constituents.

## 4. Discussion

Heavy-ion beam irradiation is increasingly acknowledged as a potent physical mutagenesis method for enhancing Chinese medicinal materials and crops, characterized by a high mutation rate and consistent inheritance of mutant traits [31,32]. Its utilization in medicinal plants shows potential for improving the yield of valuable secondary metabolites. In this investigation, we effectively utilized ^12^C^6+^ heavy-ion irradiation to create two unique, phenotypically stable, homozygous mutant lines (NZ1 and NZ2) of *Origanum vulgare* L. Our comprehensive transcriptomic and metabolomic analysis systematically unraveled the molecular mechanisms behind the irradiation-induced changes, offering new insights into the regulation of terpenoid biosynthesis, specifically the production of the key compounds carvacrol and thymol.

The phenotypic characterization of the mutants revealed significant alterations in plant architecture and biomass accumulation. Previous studies have shown that among the plant mutants obtained through heavy-ion radiation mutagenesis technology, Arabidopsis mutants exhibit multiple variant characteristics in leaves, stems, flowers or throughout the life cycle [33,34,35], while potato and chili pepper mutants show phenotypic differences in structure, development, leaves, flowers and fruits [36,37]. In this study, the contrasting phenotypes of NZ1 (reduced height, increased leaf biomass) and NZ2 (slightly increased height) suggest that heavy-ion irradiation induced diverse genetic lesions affecting different aspects of growth regulation (Figure 1). Such morphological variations are common consequences of radiation-induced genomic alterations, which can disrupt phytohormone homeostasis or cell cycle genes. For instance, in lotus flowers, mutants that can delay aging have been created by using heavy ion mutagenesis technology. In fact, this is because it affects the regulation of multiple hormones, specially making them no longer sensitive to ethylene [38]. Furthermore, the significantly higher SPAD in NZ1 indicates potential improvement in photosynthetic pigment density, a trait often associated with improved metabolic efficiency. Just as in the case of Scenedesmus quadricauda mutants induced by heavy-ion beam irradiation, the photosynthetic properties are affected through changes in central carbon metabolism, ultimately leading to two mutants’ opposite biomass accumulation [39]. These divergent phenotypes provided a solid foundation for investigating the corresponding molecular and metabolic changes.

Terpenoids are a large and diverse family of secondary metabolites that have gained more attention from chemists and pharmacologists for their intriguing skeletons and broad biological activities, including anti-inflammatory, antitumoral, antimicrobial, and anti-viral effects [40,41]. In this study, metabolomic profiling unequivocally identified terpenoids as the most abundant class of volatile organic compounds, accounting for 69.35% of the total detected metabolites (Figure 2). This predominance underscores the central role of terpenoid metabolism in oregano’s chemical composition and its particular sensitivity to heavy-ion irradiation stress. The significant number of DAVs, especially those enriched in terpenoid biosynthesis pathways, directly links the physical mutagen to a reprogramming of secondary metabolism [42]. This observation aligns with findings in Astragalus mongholicus where mutagenic treatments significantly modulated amino acid metabolism and secondary metabolite biosynthesis pathways.

Previous studies have demonstrated that multiple signaling pathways, including the ubiquitin–proteasome pathway, MAPK signaling–plant, epigenomic regulatory pathways, and plant hormones, play crucial roles in regulating the accumulation of plant secondary metabolites. As essential components of regulatory networks, MAPK cascades initiate the synthesis of secondary metabolites by phosphorylating downstream substrates, such as transcription factors and key enzymes [43]. For instance, in *Catharanthus roseus* L., the CrMAPKKK1-CrMAPKK1-CrMAPKK3/6 cascade activates the *ORCA* gene cluster, thereby enhancing the synthesis of terpene indole alkaloids (TIAs) [44]. In *Arabidopsis thaliana*, MPK3 and MPK6 phosphorylate WRKY33, which in turn activates the expression of the phytochelatin camalexin synthesis gene (*PAD3*) [45]. In rice, the OsMKK4-OsMPK6 module regulates the chitin-induced synthesis of diterpenoid phytocannabinoids [46]. These findings underscore the essential role of the MAPK pathway in the accumulation of secondary metabolites. In this study, transcriptome analysis identified 6204 DEGs, with significant enrichment in critical pathways, including the biosynthesis of secondary metabolites and the MAPK signaling pathway in plants, which are vital to the genomic response to ^12^C^6+^ heavy-ion radiation. These results indicate that heavy ion radiation alters the synthesis of secondary metabolites in the mutants by affecting genes within the oregano MAPK signaling–plant pathway, consistent with the findings of previous studies. Furthermore, transcription factors are pivotal in regulating gene expression by binding to cis-acting elements within the promoters of key genes associated with secondary metabolism. They function as essential intermediaries that link upstream signaling pathways, including MAPK and hormonal signals, to downstream metabolic processes. For example, within the WRKY family, AaGSW1 binds to the W-box to activate *CYP71AV1* and *AaORA* in Artemisia annua [47]. Additionally, WRKY33 facilitates sapogenin synthesis upon phosphorylation in *Arabidopsis thaliana* [48], while *SmWRKY34* inhibits the production of salvianolic acid and tanshinone in *Salvia miltiorrhiza* [49]. In the bHLH family, the AtTT8-GL3/EGL3-TTG1 complex activates genes responsible for anthocyanin and proanthocyanidin synthesis in *Arabidopsis thaliana* [50]. Furthermore, SmMYC2a/b interacts with the E-box element to regulate the synthesis of tanshinone and tansyolic acid in *Salvia miltiorrhiza* [51]. In the AP2/ERF family, AaERF1/AaERF2 activates *ADS* and *CYP71AV1* in *Artemisia annua*, thereby promoting artemisinin synthesis [52]. Within the MYB family, AtPAP1/PAP2 activates genes involved in anthocyanin synthesis [53]. Moreover, AaMYC2, a MYB-related factor, binds to the *AaGSW1* promoter to enhance artemisinin production in *Artemisia annua*, while PgMYB1 inhibits ginsenoside synthesis in *Panax ginseng* [54]. Additionally, *AtMYBL2* suppresses anthocyanin accumulation by disrupting the MBW complex [55]. This study identified several transcription factors via transcriptome analysis, including prominent members of the *WRKY*, *bHLH*, and *ERF* families among the DEGs. These transcription factors are acknowledged as regulators of plant specialized metabolism and stress responses, and their differential expression likely contributes to the observed alterations in metabolic pathways in response to heavy-ion irradiation.

A central finding of this study is the elucidation of the molecular framework that governs terpenoid biosynthesis in response to heavy-ion irradiation. We identified critical DEGs encoding enzymes in both the MVA and MEP pathways. Notably, *HMGR* and *MVD* are involved in the MVA pathway, while *DXS* and *HDR* are key components of the MEP pathway. These enzymes provide the universal precursors IPP and DMAPP essential for terpenoid synthesis [56,57]. The altered expression of these genes indicates a reconfiguration of metabolic flux toward terpenoid precursors. Additionally, the identification of critical downstream genes, particularly *GPPS* and *GGPPS*, which catalyze the synthesis of GPP and GGPP, highlights specific nodes where metabolic channeling may be affected [58,59]. Our analysis identified candidate genes directly involved in the biosynthesis of the valuable monoterpenoids carvacrol and thymol. The correlation between the expression of *TPS3*, *TPS6A*, and *TPS6C* and the content of γ-terpinene, the central precursor, strongly suggests their roles as γ-terpinene synthases [60,61]. We identified cytochrome P450 genes—*CYP71D178* and *CYP71D181* associated with carvacrol, and *CYP71D10B* associated with thymol—whose expression patterns closely mirrored the accumulation of these phenolic monoterpenoids. This finding provides compelling evidence for their proposed roles in the regioselective hydroxylation of γ-terpinene at the C3 and C2 positions, respectively, which represents a critical branching point that determines the final product outcome [62,63].

Despite these findings, it is imperative to acknowledge the limitations of the present study. Most notably, while pronounced phenotypic, transcriptomic, and metabolomic alterations were observed in the NZ1 and NZ2 mutants, the exact genomic mutations caused by ^12^C^6+^ irradiation remain unidentified. It is well established that heavy-ion beams are capable of inducing large-fragment deletions, insertions, and chromosomal rearrangements, which have the potential to disrupt gene function and regulatory networks. However, in the absence of whole-genome sequencing or detailed genomic structural analysis, it is not possible to identify the specific genetic lesions responsible for the observed changes in plant architecture, terpenoid accumulation, or gene expression patterns. This discrepancy hinders our capacity to establish direct causal relationships between specific genomic alterations and the reprogramming of metabolic pathways. It is imperative that future studies employ whole-genome sequencing or fine-mapping approaches in order to ascertain the precise mutations that underpin the mutant traits and to validate the regulatory mechanisms proposed in this study.

## 5. Conclusions

In conclusion, the present study establishes that ^12^C^6+^ heavy-ion irradiation is an effective means of inducing genetic and metabolic variation in *Origanum vulgare* L. By integrating multi-omics data, the present study has systematically characterized the morphological, metabolic, and transcriptional consequences of mutagenesis, revealing a complex reprogramming of terpenoid metabolism. The identification of key structural genes and transcription factors, and most notably, the candidate genes *TPS3/6A/6C*, *CYP71D178*, *CYP71D181*, and *CYP71D10B* as potential determinants of carvacrol and thymol composition, represents a significant advance in the field. These findings provide a robust molecular foundation and valuable genetic resources for future metabolic engineering and marker-assisted breeding strategies aimed at optimizing oregano for enhanced medicinal and commercial value.

## Data Availability

The original contributions presented in this study are included in the article/Supplementary Material. Further inquiries can be directed to the corresponding author.

## Supplementary Materials

The following supporting information can be downloaded at https://www.mdpi.com/article/10.3390/cimb48010007/s1.
