# Supplementary material for: Uncovering the Molecular Response of Oregano (Origanum vulgare L.) to 12C6+ Heavy-Ion Irradiation Through Transcriptomic and Metabolomic Analyses
Source: Curr Issues Mol Biol. 2025 Dec 21;48(1):7. doi: 10.3390/cimb48010007 (PMC12839708; doi:10.3390/cimb48010007)
Supplement: Supplementary file 1 [file cimb-48-00007-s001.zip › caption.pdf]

current issues in molecular biology

Figure S1: Effects of different 12C6+ radiation doses on the germination rate, emergence rate, and survival rate of Oregano Seeds;

Figure S2: Total ionic GC-MS fngerprints in heavy-ion-induced oregano mutants (NZ1, NZ2) and WT;

Figure S3: Sample correlation heatmap;

Figure S4: GO enrichment of DEGs in NZ1 *vs* WT (A), NZ2 *vs* WT (B), and NZ2 *vs* NZ1 (C), respectively.

Table S1: Volatile metabolites identified in oregano mutants (NZ1, NZ2) and WT;

Table S2: The basic information of transcriptome data;

Table S3: The DEGs of NZ1 *vs* WT;

Table S4: The DEGs of NZ2 *vs* WT;

Table S5: The DEGs of NZ2 *vs* NZ1:

Table S6 The transcription factor in DEGs;

Table S7: Encoding gene information for enzymes involved in terpenoid biosynthesis;

Table S8: The primers used in this study.

https://doi.org/10.3390/10.3390/cimb48010007
