# Supplementary figures and images for: Uncovering the Molecular Response of Oregano (Origanum vulgare L.) to 12C6+ Heavy-Ion Irradiation Through Transcriptomic and Metabolomic Analyses
Source: Curr Issues Mol Biol. 2025 Dec 21;48(1):7. doi: 10.3390/cimb48010007 (PMC12839708; doi:10.3390/cimb48010007)

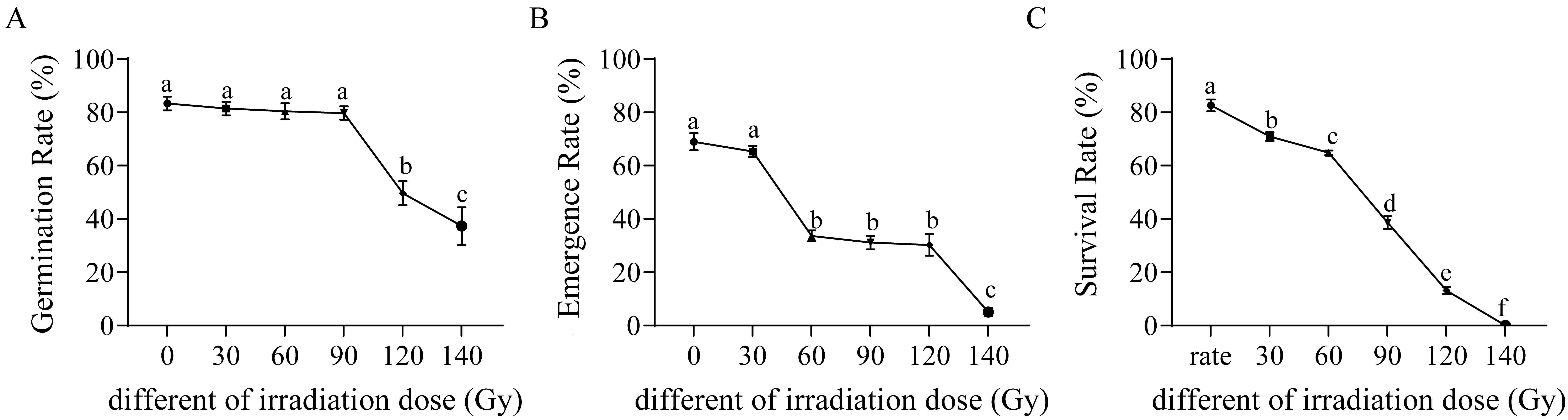

Supplement: Supplementary file 1 [file cimb-48-00007-s001.zip › Figure S1.jpg]

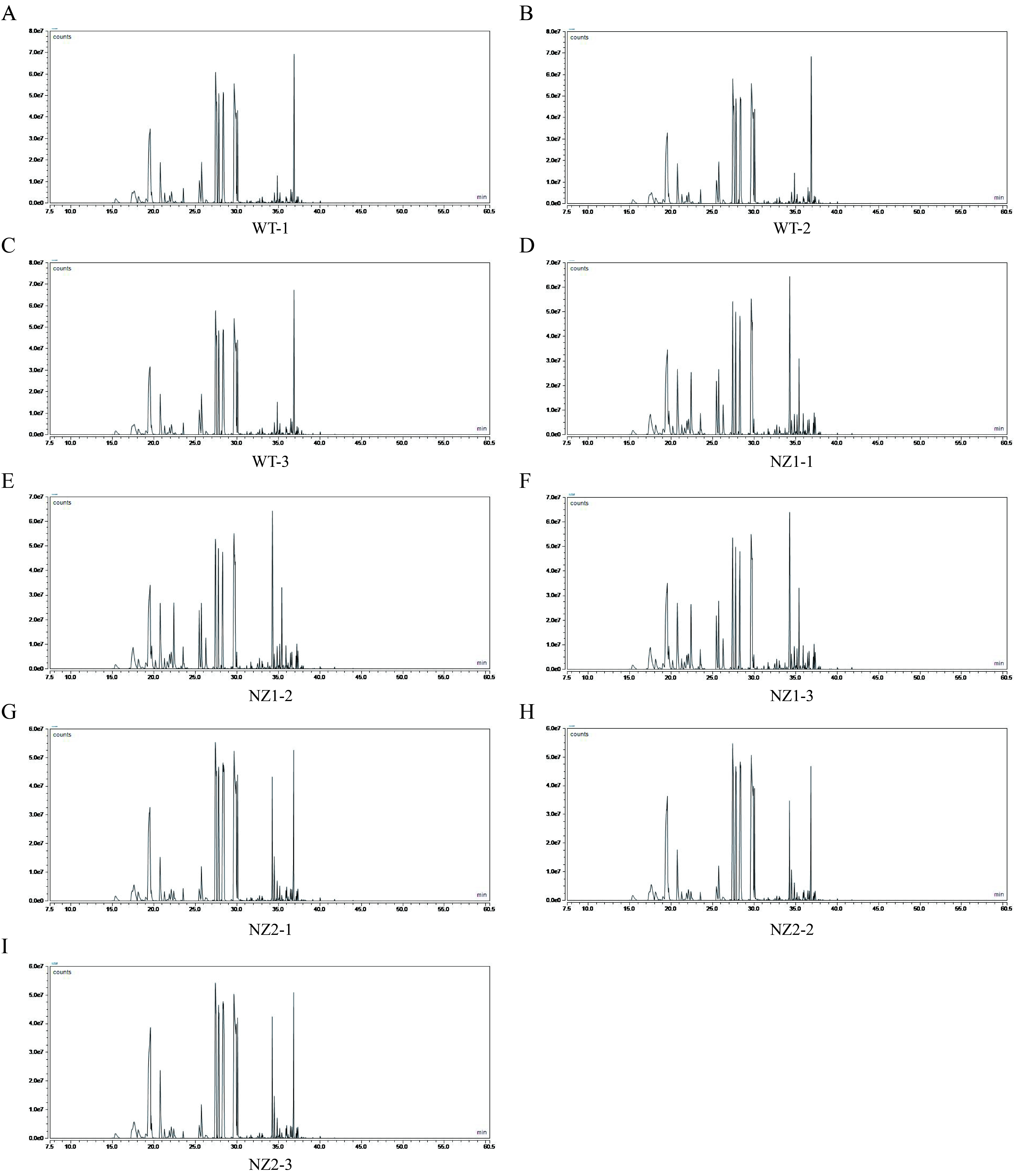

Supplement: Supplementary file 1 [file cimb-48-00007-s001.zip › Figure S2.jpg]

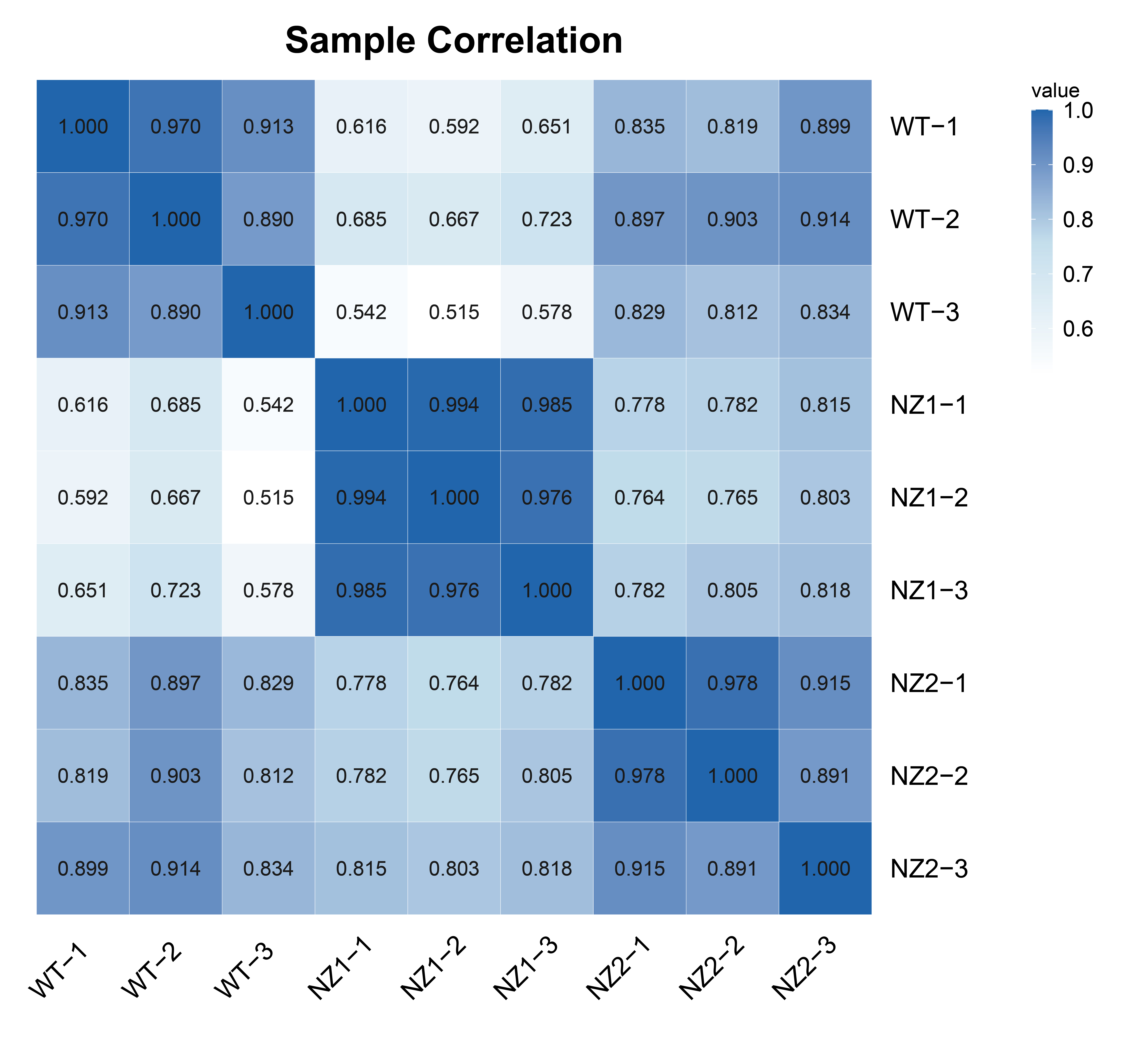

Supplement: Supplementary file 1 [file cimb-48-00007-s001.zip › Figure S3.jpg]

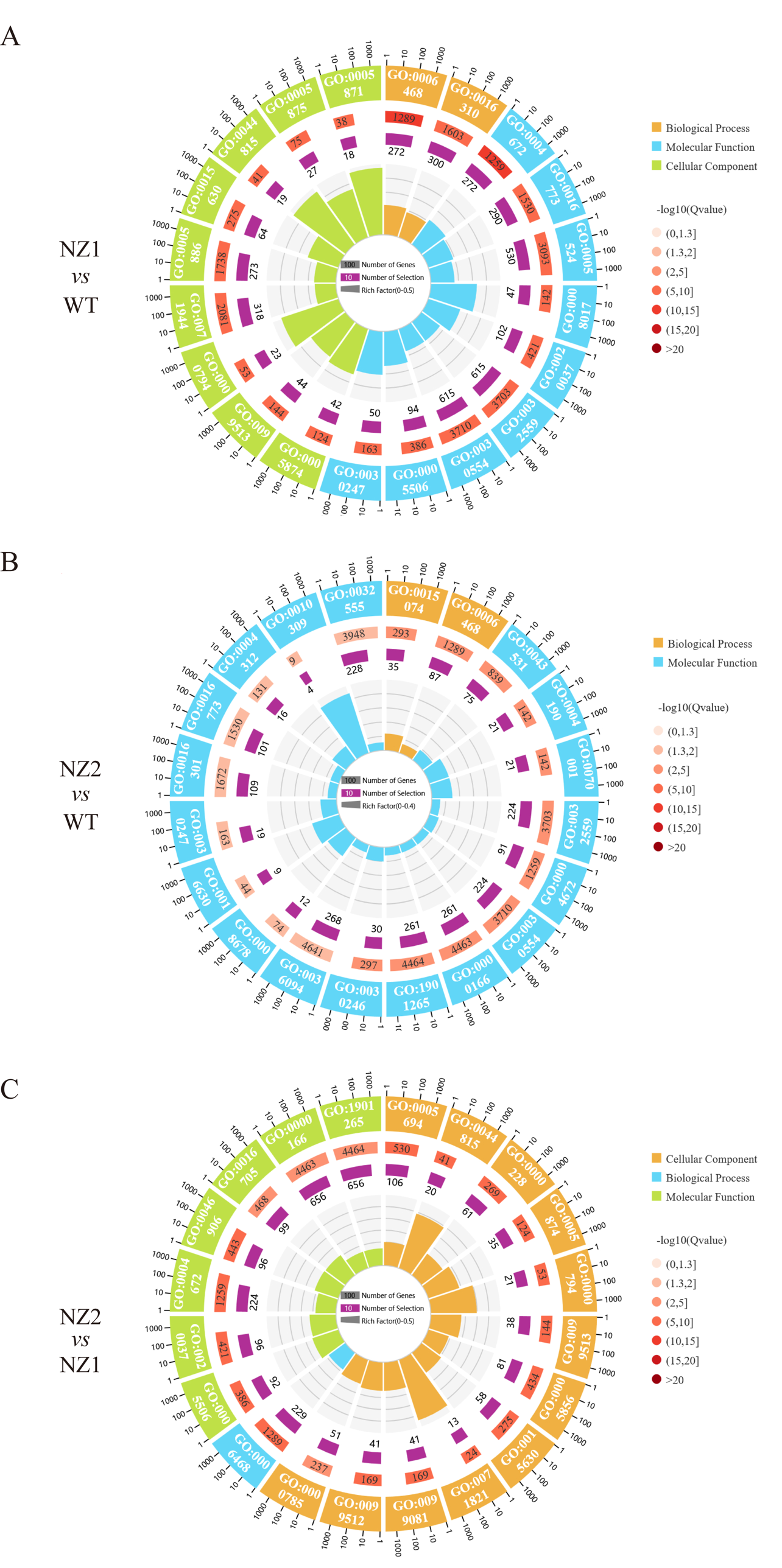

Supplement: Supplementary file 1 [file cimb-48-00007-s001.zip › Figure S4.jpg]
